# A Dual Anti-Inflammatory and Anti-Proliferative 3-Styrylchromone Derivative Synergistically Enhances the Anti-Cancer Effects of DNA-Damaging Agents on Colon Cancer Cells by Targeting HMGB1-RAGE-ERK1/2 Signaling

**DOI:** 10.3390/ijms23073426

**Published:** 2022-03-22

**Authors:** Sei-ichi Tanuma, Takahiro Oyama, Miwa Okazawa, Hiroaki Yamazaki, Koichi Takao, Yoshiaki Sugita, Shigeru Amano, Takehiko Abe, Hiroshi Sakagami

**Affiliations:** 1 Department of Genomic Medicinal Science, Research Institute for Science and Technology, Organization for Research Advancement, Tokyo University of Science, Noda 278-8510, Chiba, Japan; takahiro.oyama@hinoki.co.jp (T.O.); miwa.okazawa@rs.tus.ac.jp (M.O.); hymanami@gmail.com (H.Y.); 2 Research Institute of Odontology (M-RIO), School of Dentistry, Meikai University, Sakado 350-0283, Saitama, Japan; shigerua@dent.meikai.ac.jp (S.A.); sakagami@dent.meikai.ac.jp (H.S.); 3 Hinoki Shinyaku Co., Ltd., Chiyoda-ku 102-0084, Tokyo, Japan; mugino.abe@hinoki.co.jp; 4 Department of Pharmaceutical Sciences, Faculty of Pharmacy and Pharmaceutical Sciences, Josai University, Sakado 350-0295, Saitama, Japan; ktakao@josai.ac.jp (K.T.); sugita@josai.ac.jp (Y.S.)

**Keywords:** inflammation, tumor microenvironment, carcinogenesis, receptor for advanced glycation end-products, high-mobility group box 1, extracellular signal-regulated kinases 1 and 2, 3-styrylchromone, DNA damage

## Abstract

The current anti-cancer treatments are not enough to eradicate tumors, and therefore, new modalities and strategies are still needed. Most tumors generate an inflammatory tumor microenvironment (TME) and maintain the niche for their development. Because of the critical role of inflammation via high-mobility group box 1 (HMGB1)–receptor for advanced glycation end-products (RAGE) signaling pathway in the TME, a novel compound possessing both anti-cancer and anti-inflammatory activities by suppressing the HMGB1-RAGE axis provides an effective strategy for cancer treatment. A recent work of our group found that some anti-cancer 3-styrylchromones have weak anti-inflammatory activities via the suppression of this axis. In this direction, we searched such anti-cancer molecules possessing potent anti-inflammatory activities and discovered 7-methoxy-3-hydroxy-styrylchromone (**C6**) having dual suppressive activities. Mechanism-of-action studies revealed that **C6** inhibited the increased phosphorylation of extracellular signal-regulated kinases 1 and 2 (ERK1/2) under the stimulation of HMGB1-RAGE signaling and thereby suppressed cytokine production in macrophage-like RAW264.7 cells. On the other hand, in colorectal cancer HCT116 cells, **C6** inhibited the activation of ERK1/2, cyclin-dependent kinase 1, and AKT, down-regulated the protein level of XIAP, and up-regulated pro-apoptotic Bax and caspase-3/7 expression. These alterations are suggested to be involved in the **C6**-induced suppression of cell cycle/proliferation and initiation of apoptosis in the cancer cells. More importantly, in cancer cells, the treatment of **C6** potentiates the anti-cancer effects of DNA-damaging agents. Thus, **C6** may be a promising lead for the generation of a novel class of cancer therapeutics.

## 1. Introduction

Three lines of evidence promote the search for a new type of anti-cancer agent possessing a higher anti-inflammatory activity, targeting high-mobility group box 1 (HMGB1)–receptor for advanced glycation end-products (RAGE) signaling pathway in the tumor microenvironment (TME). First, RAGE initiates downstream pro-inflammatory signaling pathways, which are involved in the inflammatory processes seen in tumors [1,2,3]. Furthermore, the enhanced expression of RAGE has been detected in many human cancers, including brain, breast, lung, colon, prostate, and ovary [4,5,6,7]. Interestingly, there is very low or no RAGE expression in normal tissues [8,9]. These epidemiological and cellular-molecular studies showed a tight correlation between inflammation via the RAGE axis and carcinogenesis [10,11,12]. In most solid tumors, stromal cells induce cancer-associated fibroblasts (CAFs) and vascular endothelial cells and recruit a variety of supporting immune cells, such as tumor-associated macrophages (TAMs) and myeloid-derived suppressor cells (MDSCs), generating an inflammatory and immunosuppressive TME [13,14,15,16,17,18,19,20,21,22,23]. The co-operation of cancer cells and supporting immune cells facilitates tumor development via the RAGE-activated expression of inflammatory cytokines, such as interleukin (IL)-6, IL-1β, tumor necrosis factor-α (TNF-α), other cytokines, chemokines, and exosomal microRNA [24,25,26,27], which, in turn, trigger the release of pro-inflammatory RAGE ligands, including HMGB1 and S100 family proteins [28,29,30,31].

Second, under conditions of cell death or stress, HMGB1, which is a ubiquitous chromosomal protein that binds DNA and transcription factors and regulates chromatin structure and function [32,33], is passively or actively released into the extracellular milieu, where it can act as a key mediator; it can bind to cell surface receptors, such as RAGE, Toll-like receptor (TLR)4, and TLR2, thereby activating inflammatory cells and promoting their proliferation and functional maturation [34,35,36,37]. HMGB1 is one of the most abundant RAGE ligands in the inflammatory TME, and overexpression of HMGB1 in cancer cells is near-universal in solid tumors [38]. The serum level of HMGB1 is significantly associated with tumor size, depth of invasion, and lymph node metastasis [39,40]. Additionally, HMGB1 released in an environment of chronic inflammation induces various inflammatory cytokines and chemokines that drive CAFs, TAMs, and MDSCs to generate the inflammatory and immunosuppressive TME [19,38,41,42,43,44]. Indeed, MDSCs secrete HMGB1 and make up the immunosuppressive TME, which protects cancer cells from immune detection and promotes cancer survival and progression [45]. Furthermore, blockade of HMGB1 by using HMGB1-blocking antibodies inhibits tumor growth [46,47,48]. These observations suggest that the HMGB1-RAGE axis plays a critical role in immune responses due to its ability to mediate inflammation and is involved in sustained inflammation and tumor progression [8,48,49,50,51].

Finally, pentoxifylline and thalidomide, inhibitors of tumor necrosis factor (TNF)-α synthesis, enhanced the anti-tumor action of anti-cancer drugs [52,53]. Additionally, papaverine, which is an opium alkaloid antispasmodic drug used primarily for treating visceral spasm and vasospasm [54], had both anti-inflammatory and anti-cancer activities via inhibition of RAGE function [54,55,56,57]. Indeed, papaverine suppressed the HMGB1-RAGE inflammatory signaling pathway and thereby attenuated mortality in a cecal ligation and puncture-induced sepsis mouse model [54]. Furthermore, papaverine could cancel the HMGB1-elicited proliferation of human glioblastoma cell lines, both temozolomide (TMZ)-sensitive U87MG and TMZ-resistant T98G cells [56]. The combination of papaverine and TMZ significantly delayed tumor growth in a U87MG xenograft mouse model [57]. Interestingly, we recently found that some 3-styrylchromone derivatives, which have already been identified to have anti-cancer activities [58], have weak anti-inflammatory activities via the suppression of HMGB1-RAGE [59] and lipopolysaccharide-Toll-like receptor (TLR)4 [60] signaling pathways in macrophage-like RAW264.7 cells. Thus, it is expected that papaverine-mimetic 3-styrylchromone derivatives, which possess both potent anti-inflammatory and anti-cancer activities targeting the HMGB1-RAGE axis, may become valuable leads for developing a new type of anti-cancer pharmaceutical.

In this direction, we attempted to search for such dual inhibitors of 3-styrylchromone derivatives. Here, we present the first finding concerning the discovery of a potent dual inhibitor, 7-methoxy-3-(4-hydroxyl)-styrylchromone (**C6**): it potently inhibited cytokine production in macrophage-like RAW264.7 cells and induced cell cycle arrest and apoptosis in colorectal cancer HCT116 cells via suppression of HMGB1-RAGE-ERK1/2 signaling pathways. Of note, **C6** led to down-regulation of the activation of cyclin-dependent kinase 1 (CDK1) and AKT, decreased the protein level of anti-apoptotic XIAP but not c-IAP1, and c-IAP2, and increased expression of pro-apoptotic Bax and caspase-3/7. More importantly, the combination of **C6** and DNA-damaging agents, methyl methanesulfonate (MMS), etoposide (VP-16), and irinotecan (CPT-11), enhanced apoptotic cell death of cancer cells. Taken together, **C6** suppresses both inflammation and cancer cell proliferation by targeting the HMGB-RAGE-ERK1/2 signaling pathway and potentiates the anti-cancer effects of DNA-damaging agents. Therefore, as a smart molecular-mechanism-based combination strategy for DNA-directed cancer therapy, the use of **C6** combined with DNA-damaging agents may be valuable to inducing apoptosis in cancer cells. Furthermore, our present study suggests that **C6** is a useful probe to explore the linking of TME to carcinogenesis.

## 2. Results

### 2.1. Discovery of a Papaverine-Mimetic 3-Styrylchromone Derivative

To search a papaverine 3D pharmacophore-mimetic compound, approximately 2000 compounds from our in-house chemical library (MW ranging from 100 to 700, CLogP from −1 to 8, and mainly heterocyclic compounds) underwent 3D pharmacophore similarity screening using LigandScout software. By the in silico method, only one 3-styrylchromone derivative, 7-methoxy-3-methoxy-styrylchromone (**C1**), was discovered as following the required criteria: a papaverine 3D pharmacophore fit score (3DPFS) > 0.5, MW < 500, and CLogP < 5. The 3DPFS of **C1** was calculated to be 0.51. The characteristics of **C1** are the 7-methoxy group in a chromone moiety and the methoxy group at the C4′ position of the benzene ring in a 3-styryl moiety. Interestingly, the 3D positions of the two methoxy groups and aromatic rings are similar to those of papaverine.

**Figure 1:**
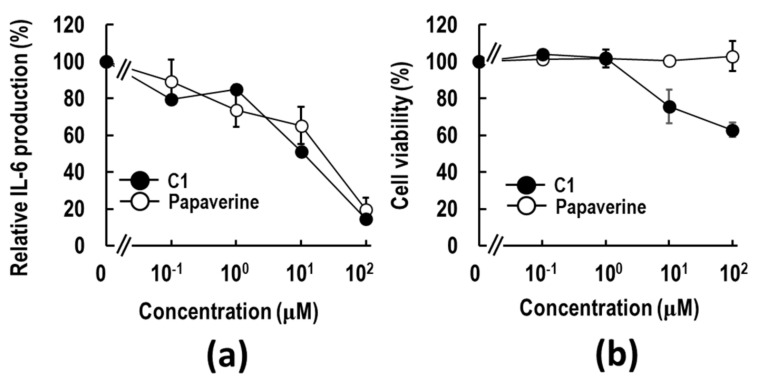
The screening of papaverine-mimetic 3-styrylchromone derivatives by 3D pharmacophore using LigandScout. (**a**) Anti-inflammatory effects of **C1** and papaverine. RAW264.7 cells were pre-treated with **C1** (closed circle) or papaverine (open circle) for 2 h, followed by stimulation by 5 μg/mL HMGB1. After 18 h of incubation, IL-6 release in the medium was measured by ELISA. (**b**) RAW264.7 cells were pre-treated with **C1** or papaverine for 2 h, followed by 5 μg/mL HMGB1 for 18 h. The cell viability was determined by the WST-8 assay as described in Section 4. The data are presented as mean ± SE of four independent experiments.

To examine whether **C1** has anti-inflammatory activity, the HMGB1-RAGE-mediated IL-6 production assay using macrophage-like RAW264.7 cells was performed. As shown in Figure 1a, **C1** was found to have a dose-dependent suppressive effect on HMGB1-stimulated IL-6 production. The 50% effective concentration (EC50) value was calculated to be 11.1 μM. The EC50 value was nearly similar to that of papaverine (EC50 = 15.7 μM). **C1** inhibited the cell viability at 10 μM. **C1** showed a slightly higher inhibitory effect than papaverine (Figure 1a), possibly due to higher cytotoxicity (Figure 1b). These results imply that a novel anti-inflammatory compound, **C1**, was discovered by the in silico 3D pharmacophore hopping of papaverine with the program in LigandScout software.

### 2.2. Three-Dimensional Pharmacophore Similarity Search for ***C1*** Mimetics

Next, to obtain C1 mimetic anti-inflammatory compounds, a 3D pharmacophore similarity search was performed using our 54 3-styrylchromone in-house libraries [61,62,63], and seven compounds were selected. As shown in Table 1, structural feature studies were conducted on two sections (A and B) of the compounds to determine the partial structures required for the potent anti-inflammatory activity. In the section labeled with A, the methoxy group at the para-position (C4′) on the benzene ring was replaced with various functional groups (compound **2**~**6**, **C2**~**C6**). In the section-labeled Part B, the methoxy group at the **C7** position of the chromone moiety was deleted (compound **7**, **C7**).

**Figure 2:**
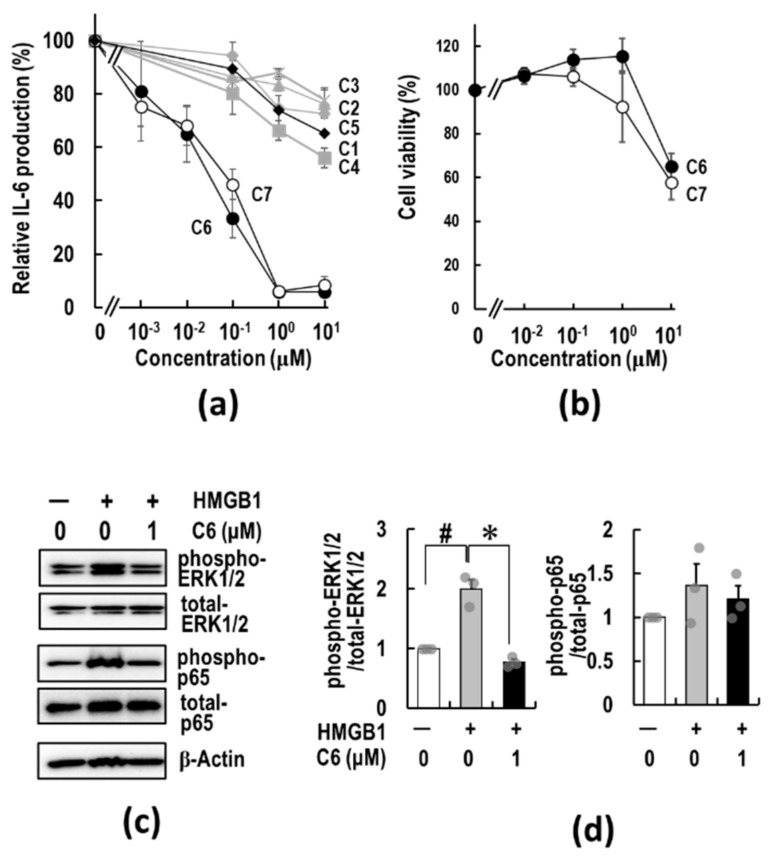
Anti-inflammatory activities of synthesized 3-styrylchromone derivatives. (**a**) Suppressive effects of 3-styrylchromone derivatives on IL-6 production in RAW264.7 cells. RAW264.7 cells were pre-treated with 3-styrylchromone derivatives for 2 h, followed by stimulation with 5 μg/mL HMGB1. After 18 h incubation, IL-6 released in the medium was measured by ELISA as described in Section 4. The data are presented as mean ± SE of four independent experiments. The symbols of the compounds are listed below; **C1** (closed diamond), **C2** (gray closed triangle), **C3** (gray cross), **C4** (gray closed square), **C5** (gray closed diamond), **C6** (closed circle), and **C7** (open circle). (**b**) Cell viability of **C6** and **C7** was determined by the WST-8 assay as described in Section 4. Data are shown as mean ± SE of three independent experiments. (c and d) RAW264.7 cells were pre-treated with or without 1 μM **C6** for 2 h and then stimulated by 5 μg/mL HMGB1. After 15 min incubation, the phosphorylation of ERK1/2 and NF-κB (p65) was measured via Western blotting as described in Section 4. β-Actin served as a loading control. The panels depicting typical immunoblots (**c**) and the relative ratio of each band intensity of ERK1/2 (left) and p65 (NF-κB) (right) were determined by densitometric analysis (**d**). Data are expressed as mean ± SE of three independent experiments. The significance was tested by the Student’s *t*-test (# or * *p* < 0.05 for non-treated group vs. HMGB1 or HMGB1-treated group vs. **C6**-treated group, respectively). The original image of Figure 2c is shown in Supplementary Figure S1.

The seven compounds were examined for their anti-inflammatory activities by HMGB1-stimulated IL-6 production assay using RAW264.7 cells. Figure 2a shows the representative titration curves of the suppression of IL-6 production by treatment with these 3-styrylchromone derivatives. The EC50 values of **C1**, **C2**, **C3**, **C4**, **C5**, **C6**, and **C7** were calculated to be 11.1, > 10, > 10, > 10, > 10, 0.018, and 0.020 μM, respectively, from the titration curves. Of the compounds tested, **C6** (7-methoxy-3-hydroxy-styrylchromone) showed the most potent suppressive effect on IL-6 production in HMGB1-stimulated RAW 264.7 cells. Namely, when the methoxy group in the styryl moiety of **C1** was substituted for the hydroxy group (**C6**), the anti-inflammatory activity was significantly increased. The suppressive activity of **C6** was considerably higher than that of the fluoride- (**C2**), chloride- (**C3**), dimethylamine- (**C4**), and hydrogen- (**C5**) substituted compounds. Importantly, the 7-methoxy-deleted **C7** had the same high suppressive activity as **C6**. Additionally, **C6** and **C7** showed no obvious cytotoxic effects on RAW 264.7 cells at concentrations of up to 1 μM (Figure 2b). At a higher concentration of 10 μM, which is about three orders of magnitude higher than the EC50 values of **C6** and **C7** (Table 1), cytotoxicities appeared in the macrophages. These results suggest that the substituent of the hydroxyl group at the para-position of the benzene ring in a 3-styryl moiety in **C6** and **C7** is significant in the attenuation of HMGB1-stimulated pro-inflammatory response. However, the 7-methoxy group (R1) in a chromone moiety is not always required for anti-inflammatory activity.

### 2.3. ***C6*** Exerts an Anti-Inflammatory Effect via Suppression of HMGB1-RAGE-ERK1/2 Signaling Pathway in RAW264.7 Cells

The above results indicate that the **C6** strongly suppresses HMGB1-induced pro-inflammatory responses in the macrophages by attenuating the HMGB1-RAGE pro-inflammatory signaling pathway. Through the extracellular binding of HMGB1 to RAGE, the cytoplasmic tail of RAGE interacts with key factors, which activate downstream RAGE mediators, such as ERK1/2 and c-Jun N-terminal kinase (JNK), in the regulation of pro-inflammatory cytokine production [59]. Thus, whether **C6** regulates the activation of downstream pro-inflammatory RAGE signaling, the activation of ERK1/2 was examined using HMGB1-stimulated RAW264.7 cells. As expected, **C6** effectively suppressed the increased ERK1/2 phosphorylation caused by the HMGB1 treatment for 15 min (Figure 2c,d). The ability of **C6** to engage in the regulation of transcription of cytokines in the nucleus was determined by assessing NF-κB (p65) activation. As shown in Figure 2c,d, the HMGB1 treatment for 15 min induced an increase in p65 phosphorylation, and by **C6** treatment, the increased p65 phosphorylation was suppressed. Thus, **C6** is suggested to exert anti-inflammatory effects on HMGB1-RAGE signaling by down-regulating the ERK1/2 pathway and the subsequent suppression of NF-κB activity.

### 2.4. ***C6*** Inhibits Proliferation and Induces Apoptosis in HCT116 Cells

**Figure 3:**
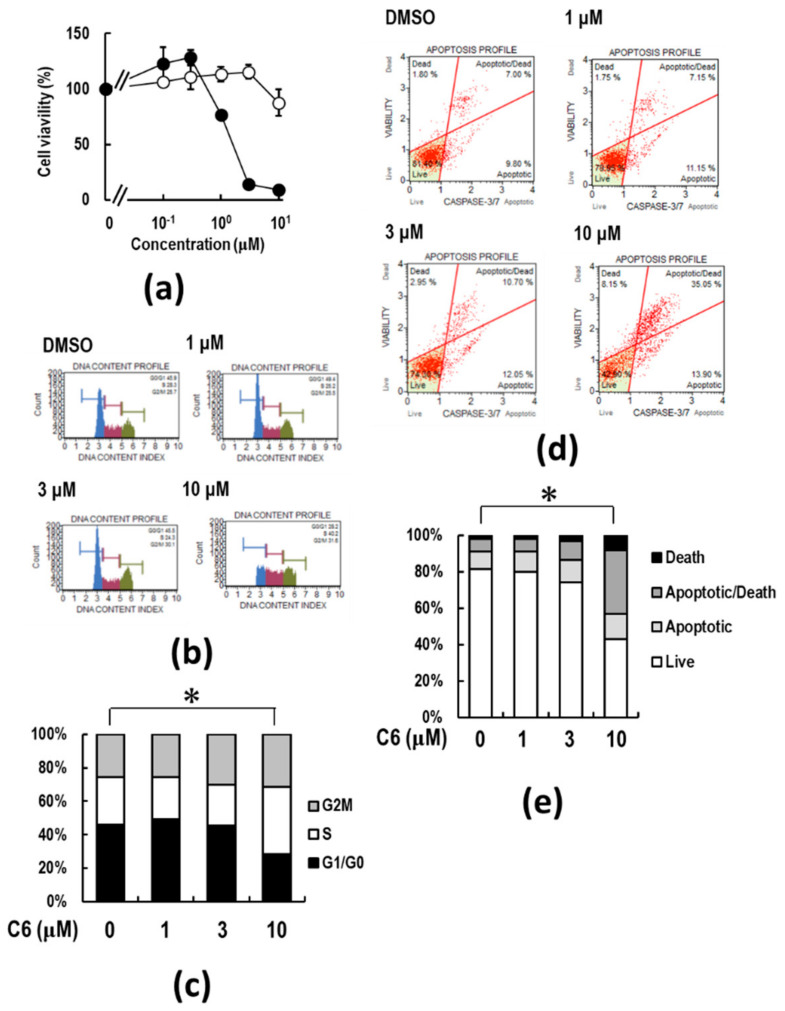
Anti-proliferative activities of **C6** and **C7** in HCT116 cells. (**a**) WST-8 cell activity assay was performed as described in Section 4. HCT116 cells were treated with 0, 0.01, 0.03, 0.1, 0.3, 1, 3, and 10 μM **C6** or **C7** for 72 h. (**b**,**c**) Cell cycle analysis was performed by flow cytometer. HCT116 cells were treated with 0 (DMSO), 1, 3, and 10 μM **C6** for 48 h. (**d**,**e**) Caspase-3/7 activity was measured by flow cytometer. HCT116 cells were treated with 0 (DMSO), 1, 3, and 10 μM **C6** for 48 h. Population analyses of apoptotic cells were performed by a flow cytometer. The horizontal and vertical axes indicate the caspase-3/7 activity from the signal of the cleavage of the fluorescent dye-conjugate substrate and cell death from the signal of 7-AAD-positive cells, respectively. * The distributions of population of cell cycle and cell status (caspase-3/7 activation) were compared by χ^2^ goodness-of-fit test with a significance level set as α = 0.05.

We next examined whether **C6** and **C7** have anti-cancer activity. A representative human cancer cell line, colorectal carcinoma HCT116 cells, was used to investigate the effects of **C6** and **C7** on cancer cell proliferation. Interestingly, treatment with **C6** for 72 h significantly dose-dependently reduced the viability of HCT116 cells with an EC50 value of 0.84 μM, while **C7** had little effect on cell viability up to 10 μM (Figure 3a). To evaluate the anti-proliferation activity of **C6**, cell cycle analysis was performed. Notably, **C6** treatment significantly decreased the proportion of the G1 phase, whereas it increased the proportion of S and G2/M phases compared to that in the control cells (Figure 3b,c).

**Figure 4:**
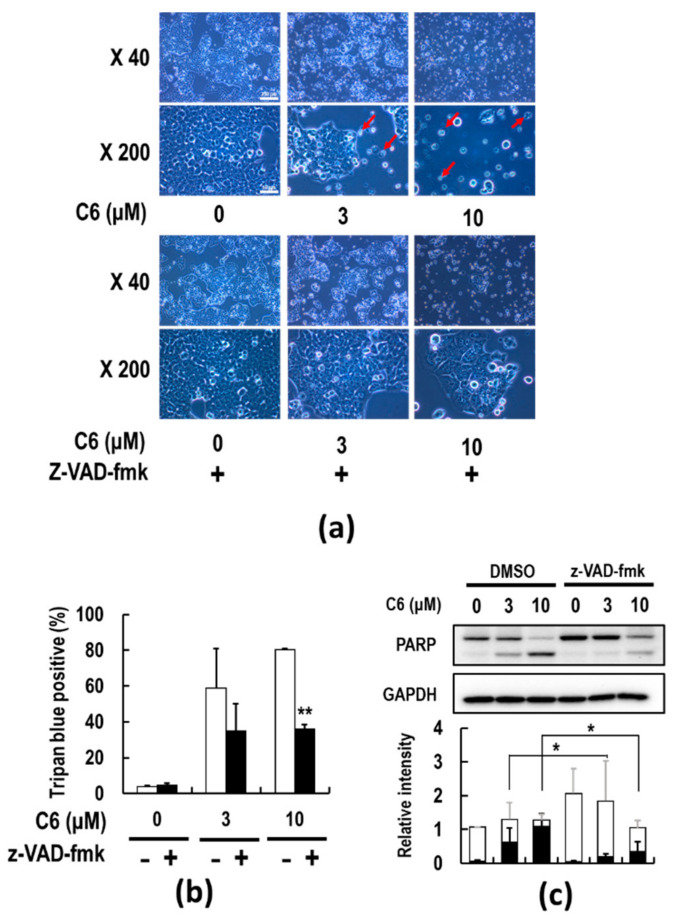
The effect of the pan-caspase inhibitor z-VAD-fmk on **C6**-induced apoptosis. HCT116 cells were pre-treated with or without 20 μM z-VAD-fmk for 2 h and followed by treatment with 0, 3, and 10 μM **C6** for 72 h (**a**,**b**) or 48 h (**c**). (**a**) Microscopic images of HCT116 cells. The arrows indicate the apoptotic cells. (**b**) Dead cells were counted by the trypan blue dye exclusion. Approximately 1000 trypan blue positive or negative cells were counted at each point. ** *p* < 0.01 by Student’s *t*-test. (**c**) Immunoblot images indicate PARP cleavage; the black and white bars represent full-length and cleaved PARP, respectively. The significance was tested by the Student’s *t*-test (* *p* < 0.05). The original image of Figure 4c is shown in Supplementary Figure S2.

To examine the induction of apoptosis, 7-AAD and caspase-3/7 analyses were performed by a flow cytometer (Figure 3d). The percentage of early apoptotic death ofHCT116 cells [7-AAD (+)/Caspase-3/7 (+)] after 48 h of treatment with 10 μM **C6** increased by approximately 40% (Figure 3d,e). Next, the effect of the pan-caspase inhibitor z-VAD-fmk on caspase-3/7 activity was examined following **C6** treatment. As a result, HCT116 cells began to shrink and detach after 48 h of **C6** treatment, and z-VAD-fmk treatment blocked the morphological changes (Figure 4a). Cell death detected by trypan blue dye exclusion assay increased to approximately 60 and 80% by 3 and 10 μM **C6** treatment, respectively, after 72 h treatment (Figure 4b). Additional treatment with z-VAD-fmk suppressed cell death (Figure 4b). In addition, the **C6**-induced cleavage of a hallmark of apoptosis, poly(ADP-ribose) polymerase (PARP), in a fragment of 89 kDa, which is a main substrate of caspase-3/7, was suppressed by co-treatment with z-VAD-fmk (Figure 4c). Thus, **C6**-induced cell death by apoptosis of HCT116 cells was suggested to be caspase-dependent.

### 2.5. ***C6*** Suppresses HMGB1-RAGE Signaling by Inhibiting the Activation of ERK 1/2 in HMGB1-Stimulated HCT116 Cells

**Figure 5:**
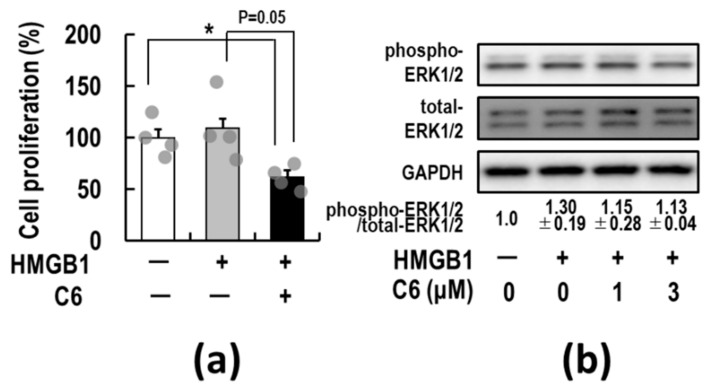
Suppressive effect of **C6** on the HMGB1-stimulated activation of ERK1/2 in HCT116 cells. (**a**) HCT116 cells were pre-treated with 3 μM **C6** for 2 h and then stimulated with 10 μg/mL HMGB1 or vehicle (phosphate-buffered saline) for 72 h. The relative cell proliferation rate (%) was measured using trypan blue exclusion assay as described elsewhere in Section 4 and represents the mean ± SE of four independent experiments. *p* values were calculated against vehicle control with the Student’s *t*-test. * *p* < 0.05 was accepted as a significant difference. (**b**) HCT116 cells were pre-treated with 3 μM **C6** at indicated concentrations for 2 h and then stimulated with HMGB1 (10 μg/mL) for 30 min. Cell lysates were subjected to Western blotting using phospho-ERK1/2 and total ERK1/2 antibodies as described in Section 4. GAPDH served as a loading control. The panels depicted a typical immunoblot. The values are the ratios of each phospho-ERK1/2 to total-ERK1/2 band intensities calculated by densitometric analysis. Values represent the mean ± SE of three independent experiments. The original image of Figure 5b is shown in Supplementary Figure S3.

The above observations indicate that the mechanisms underlying the decrease in cell viability by **C6** treatment are correlated with the induction of apoptosis. As HMGB1 promotes cancer cell growth by acting as an autocrine factor in human glioblastoma T98G and U87MG cells [56,57], we examined whether supplemental HMGB1 stimulates the proliferation of HCT116 cells. As shown in Figure 5a, a slight stimulatory effect of HMGB1 on HCT-116 cells was observed. It was noted that **C6** suppressed the HMGB1-stimulated proliferation of HCT116 cells to below the basal level. These observations imply that HCT116 cells are continuously activated by various growth factors, including HMGB1, carried from fetal bovine serum (FBS) in culture medium.

Through the binding of HMGB1 to RAGE, the cytoplasmic tail of RAGE interacts with the key mediator kinase ERK 1/2, activating downstream mediators for cell proliferation [60,64,65]. Thus, whether **C6** suppresses the activation of ERK1/2 was first examined in HMGB1-stimulated HCT116 cells. The level of phosphorylation of ERK 1/2 was determined by Western blotting (Figure 5b). Compared with those of the HMGB1-non-treated control group, the phosphorylation levels of ERK 1/2 were increased approximately 1.3-fold in the HMGB1-stimulated group. There were some basal levels of phosphorylated ERK1/2 in HMGB1-non-stimulated HCT116 cells, suggesting constitutive background activation of ERK1/2 via various growth factors carried from FBS in the culture medium. Importantly, in the HMGB1-stimulated group, the increased phosphorylation levels of ERK 1/2 decreased to near the basal level by 1 and 3 μM **C6** treatment. This decrease was suggested to be at least in part involved in the **C6**-induced decrease of cell proliferation.

### 2.6. Molecular Mechanism of Action of ***C6*** in HMGB1-Stimulated HCT116 Cells

**Figure 6:**
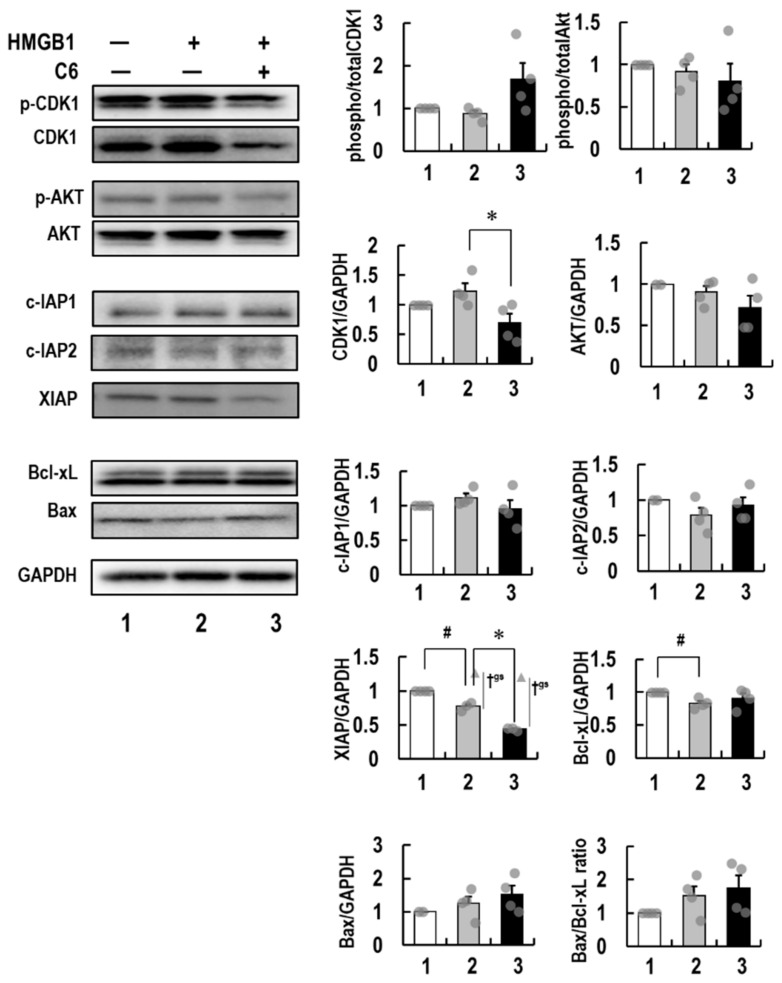
The effect of **C6** on cell-cycle-, proliferation-, and apoptosis-related proteins in HMGB1-stimulated HCT116 cells. (**left blotting panels**) Immunoblot analyses of phospho-CDK1 and -AKT and the protein levels of IAPs, Bcl-xL, Bax, and GAPDH were performed using protein lysates from HCT116 cells pre-treated with 3 μM **C6** for 2 h with 10 μg/mL HMGB1 or vehicle for 72 h, as described in Section 4. GAPDH served as a loading control. (**right**) The relative ratios were presented as the bar of at least three independent experiments of each protein band intensity determined by densitometric analysis. The significance was tested by the Student’s *t*-test (# or * *p* < 0.05). Smirnov–Grubbs test was used to remove outlier data. The significance level was set as α = 0.05. The outlier data were expressed as a triangle plot and specified as †^gs^. The original image of Figure 6 is shown in Supplementary Figure S4.

The above results indicate that the activation of ERK 1/2 by the interaction of HMGB1 with RAGE was inhibited by **C6** treatment, resulting in suppression of downstream signaling for cell proliferation and apoptosis. Thus, the expression levels of various critical factors affecting the cell cycle, proliferation, and apoptosis in cancer cells were investigated. HTC116 cells stimulated by 10 μg/mL HMGB1 were used to examine whether **C6** affected the expression of cell proliferative (CDK1 and AKT), anti-apoptotic (IAPs, Bcl-2, and Bcl-xL), and pro-apoptotic (Bax) molecules. Intriguingly, immunoblot analyses showed that phospho-CDK1 and phospho-AKT were detected at high levels in HMGB1-stimulated and -non-stimulated HCT116 cells. Importantly, **C6** decreased phospho-AKT, as well as the total protein levels of CDK1 and AKT, to below the basal levels of HMGB1-non-treated HTC116 cells (Figure 6). Of note, **C6** decreased the XIAP protein level to below the basal level, compared with c-IAP1 and c-IAP2. The expression of anti-apoptotic Bcl-xL was not influenced by **C6** treatment. The expression of Bcl-2 was hard to detect in HCT116 cells. In contrast, **C6** increased the expression of pro-apoptotic Bax, although the increased ratio of Bax/Bcl-xL was not significant. These results indicate that **C6** reduces the level of anti-apoptotic XIAP but not c-IAP1 and c-IAP2 and induces the expression of pro-apoptotic Bax via the suppression of the HMGB1-RAGE-ERK1/2 axis. Thus, these alterations induced by **C6** are suggested to be involved in the anti-proliferation and pro-apoptotic processes.

### 2.7. ***C6***, along with DNA-Damaging Agents, Synergistically Induces Apoptosis

**Figure 7:**
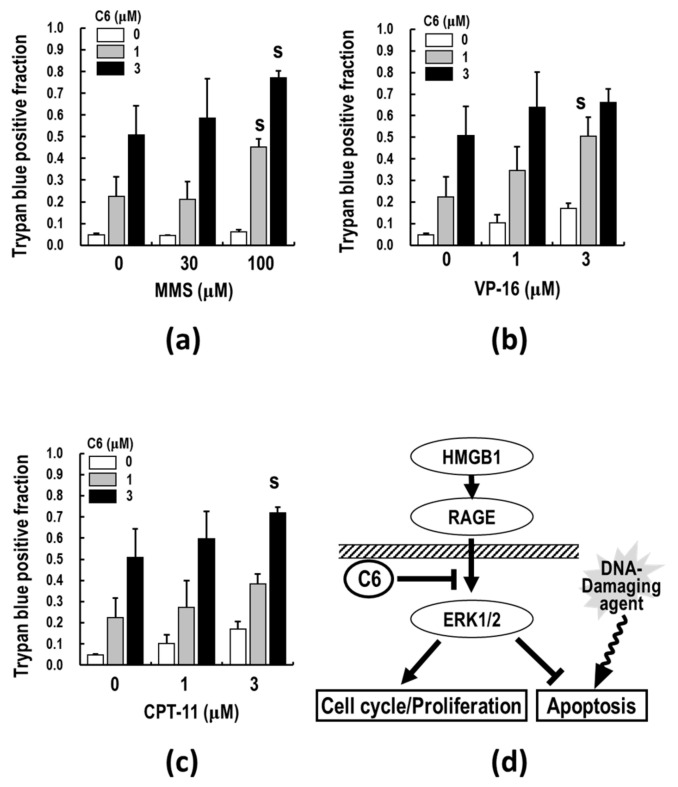
The effect of **C6** on cell death induction along with DNA-damaging agents in HCT116 cells. HCT116 cells were co-treated with **C6** and MMS (**a**), VP-16 (**b**), or CPT-11 (**c**) at the indicated concentrations for 48 h. Cell death was measured by trypan blue dye exclusion as described in Section 4. The synergy analysis of the combined effects was performed by excess-over-Bliss additivism (EOBA) analyses. Data are presented as mean ± SE of three independent experiments. S represents a synergistic effect. (**d**) Schematic illustrating the suppressive effects of **C6** on cell cycle/proliferation and apoptosis via HMGB1-RAGE-ERK1/2 axis and the combination effects of DNA damaging agents on apoptosis induction in cancer cells.

Since the above analyses of apoptosis-related proteins revealed that **C6**-induced apoptosis depends on XIAP and Bax but not Bcl-xL, the combination effects of **C6** with DNA-damaging agents (MMS, VP-16, and CPT-11) were examined. Interestingly, the results show that co-treatment of **C6** with MMS synergistically induced cell death of HTC116 cells (Figure 7a). Trypan blue positive dead cells synergistically increased by approximately 6- and 13-fold after 48 h of co-treatment with 100 μM in the presence of 1 and 3 μM **C6,** respectively, in HCT116 cells. The excess-over-Bliss additivism (EOBA) values are 0.18 and 0.23, respectively, whose value indicates synergy if it becomes more than 0.1 [66]. A single treatment with 1 and 3 μM **C6** induced cell death by approximately 3- and 7-fold at 48 h. In addition, co-treatment of **C6** with VP-16 (1 μM **C6**/3 μM VP-16: EOBA = 0.15) (Figure 7b) or CPT-11 (3 μM **C6**/3 μM CPT-11: EOBA = 0.13) (Figure 7c) enhanced the appearance of dead cells. However, in the combinations of low-dose **C6** and the DNA-damaging agents, synergistic effects were not observed. Thus, the co-treatment of **C6** with DNA-damaging agents, MMS, CPT-11, or VP-16, in appropriate conditions, synergistically induces cell death, as compared to **C6** treatment alone. Taken together, these results suggest that **C6** potentiates the anti-cancer effects of DNA-damaging agents on cancer cells (Figure 7d).

## 3. Discussion

**Figure 8:**
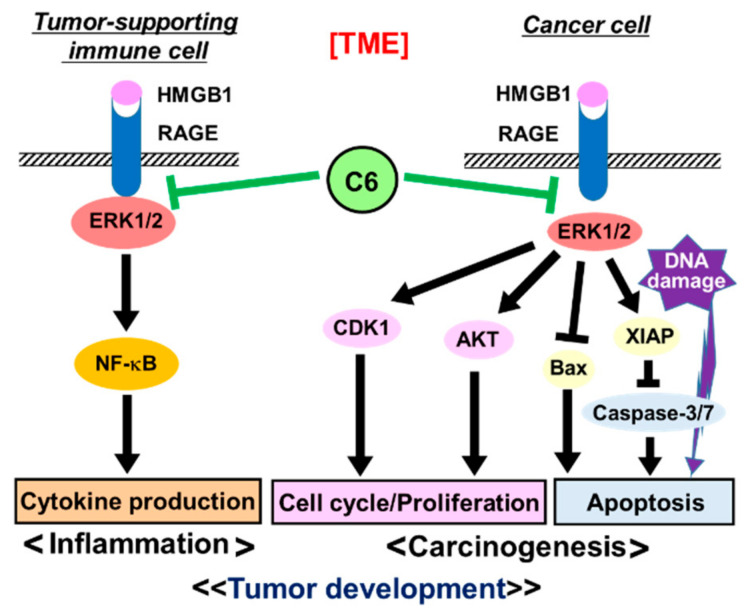
Schematic model for the anti-inflammatory and anti-proliferative effects of **C6** on tumor-supporting immune cells and cancer cells, respectively, through the HMGB1-RAGE- signaling pathway in the TME. The potential mechanisms of the suppressive effects of **C6** on cytokine production in tumor-supporting cells and cell cycle/proliferation and apoptosis in cancer cells via HNGB1-RAGE-ERK1/2 signaling pathway are shown. Additionally, the anti-cancer combination effects of **C6** and DNA-damaging agents are illustrated. The interaction of HMGB1 with RAGE up-regulates the RAGE downstream signaling pathways. Consequently, RAGE-regulated genetic programs of pro-inflammation and carcinogenesis start driven by the action of various transcription factors, such as NF-kB and AP-1. **C6** potently suppresses HMGB1-RAGE-ERK1/2 signaling and potentiates the anti-cancer effects of DNA-damaging agents on cancer cells.

The current anti-cancer treatments are not enough to eradicate tumors, and therefore, new modalities and strategies are still needed. To develop a robust system in the TME, cancer cells must recruit a variety of tumor-supporting cells from the stromal tissues, such as CAFs, TAMs, and MDSCs [17,18,19]. The activations of recruited CAFs, TAMs, and MDSCs in the TME by various factors, including HMGB1, are important physiological barriers for penetrating T and NK cells through the release of immunosuppressive cytokines and chemokines, thus making cancer cells escape from immunosurveillance [43,44]. The use of anti-inflammatory agents targeting the TME has recently gained attention as a cancer treatment option since the TME acts as an essential ingredient of cancer malignancy [40]. Furthermore, accumulating evidence has suggested a functional role for HMGB1-RAGE signaling in the linking of inflammation to carcinogenesis [41,67]. Therefore, it is of particular importance to identify a new type of anti-cancer agent suppressing not only cancer cell proliferation but also inflammation in the TME and to clarify the molecular nature of inflammation to carcinogenesis. The results of this study show that **C6**, which was discovered as a papaverine 3D pharmacophore mimetic compound (Table 1), has both potent anti-inflammatory (Figure 2) and anti-cancer (Figure 3 and Figure 4) activities by the suppression of the HMGB1-RAGE-ERK1/2 signaling pathway (Figure 5 and Figure 6), inducing apoptosis to cancer cells (Figure 8). The 3D pharmacophore-activity relationship analyses of seven 3-styrylchromone derivatives revealed the importance of the hydrogen bond donor of the hydroxy group at the benzene ring in the 3-styryl moiety and the hydrogen bond acceptor of the 7-methoxy group at the chromone moiety in **C6** for the potent suppressive effects on both inflammation in macrophages and proliferation in cancer cells. Consequently, this unique dual inhibitor of **C6** may be effective in inducing cell death by apoptosis to cancer cells and improving the immunosuppressive TME. Further studies on the dual-active agents possessing anti-cancer and anti-inflammatory activities using rigid synthesized **C6** derivatives are needed to understand the molecular mechanisms involved in the dual suppression of tumor development.

It is of particular importance to clarify the molecular pathways involved in the dual suppressive effects of **C6**. Importantly, **C6** potently suppressed the HMGB1-RAGE-mediated inflammation axis in macrophage-like RAW264.7 cells (Figure 2). Furthermore, to understand the anti-cancer effects of **C6** on the HMGB1-RAGE signaling pathway, the underlying mechanism of action of **C6** was investigated in HMGB1-stimulated colon cancer HCT116 cells (Figure 6). Through the extracellular binding of HMGB1 to RAGE, the cytoplasmic tail of RAGE interacts with the key mediator kinase ERK1/2 [60,64,65], activating downstream RAGE effectors. The activated ERK1/2 by phosphorylation are known to activate CDK1, which plays an essential role in the G2/M transition of the cell cycle [68,69,70]. Additionally, AKT regulates not only cell proliferation but also apoptosis [71]. Of note, **C6** inhibited the phosphorylation of ERK1/2 induced by HMGB1 stimuli and thereby suppressed the activation of the mediator kinases CDK1 and AKT (Figure 6), resulting in the down-regulated cell cycle/proliferation (Figure 8). The increased proportion of G2/M phases by **C6** treatment (Figure 3b and c) will not contradict the suppression of CDK1 [68,69]. Notably, the anti-apoptotic XIAP, which is a biomarker of cancers and causes apoptosis resistance to some cancer therapies [72], was decreased by **C6** treatment in HCT116 cells. In contrast, the levels of c-IAP1 and c-IAP2 were not affected by **C6** treatment. Importantly, **C6** up-regulated the level of pro-apoptotic Bax [73] but not anti-apoptotic Bcl-xL [74]. Furthermore, the activation of the apoptosis executor, caspase-3/7 was observed. Therefore, the inhibitory mechanisms of **C6** on the HMGB1-RAGE-ERK1/2 axis are considered to cause the suppression of not only inflammation response but also cancer cell proliferation, thus inducing apoptosis. However, further studies are still required to investigate if there are other underlying molecular mechanisms involved in **C6**-suppressed inflammation and cancer cell proliferation. Nevertheless, the dual suppressive effect of **C6** on macrophages and cancer cells is an important finding for developing a new type of anti-cancer drug.

More importantly, the co-treatment of **C6** with DNA-damaging agents resulted in the enhancement of cancer cell death by apoptosis (Figure 7), indicating that **C6** requires a DNA-damaging agent to exert effectively its apoptosis-inducing effect on cancer cells (Figure 8). It is conceivable that crosstalk between the **C6**-inhibiting HMGB1-RAGE signaling pathway and DNA damage may occur in alterations of gene regulation, including suppression of inflammation in the TME and cancer cell proliferation and induction of apoptosis. If the level of DNA-damaging-agent-induced DNA damages, base or sugar damages, and single- or double-strand break exceeds its repair ability, the apoptotic pathways are triggered, leading to cell death. Therefore, the **C6**-induced apoptotic pathways could enhance the apoptosis inducibility of DNA-damaging agents. Thus, the DNA repair ability of cancer cells may be the key factor of determining the potentiation of the combination of **C6** and DNA-damaging agents. Furthermore, since the dying cancer cells co-treated with **C6** and DNA-damaging agents release HMGB1 from the nucleus to the extracellular milieu and chromosomal HMGB1 is involved in DNA repair mechanisms, such as nucleotide excision repair, base excision repair, and mismatch repair [75,76,77], the DNA repair ability may be extensively reduced by the combination. **C6** could probably suppress inflammation in the TME and cytokine storm induced by the released HMGB1. These data provide convincing evidence that the co-treatment of **C6** with a DNA-damaging agent is an appropriate molecular-based combination for cancer treatment. Thus, the present findings of the potent suppressive effects of **C6** on the HMGB1-RAGE-ERK1/2 signaling pathway may give us clues for a new way to further develop novel and effective anti-cancer drugs and therapeutic strategies against various cancers, although further studies with additional cancer cell types are needed to confirm these results. In addition, further studies must determine the target(s) of the dual active **C6** to clarify the suppressive effects on CAFs, TAMs, and MDSCs and the molecular linking of chronic inflammation to carcinogenesis for in vivo application.

## 4. Materials and Methods

### 4.1. Chemicals and Reagents

Papaverine and MMS were purchased from SIGMA (St. Louis, MO, USA). VP-16 and Etoposide were purchased from LKT Lab. (St. Paul, MN, USA) and Tokyo Chemical Industry Co., Ltd., (Tokyo, Japan), respectively. Recombinant bovine HMGB1 was purchased from Chondrex Inc., (Redmond, WA, USA). Dulbecco’s Modified Eagle Medium (DMEM) and WST-8 reagent were purchased from Fujifilm Wako Pure Chemicals (Osaka, Japan). Opti-MEM^®^ and Mouse IL-6 Uncoated ELISA Kit were purchased from Thermo Fisher Scientific (Waltham, MA, USA). Anti-rabbit horseradish peroxidase (HRP)-labeled IgG antibody was obtained from GE Healthcare (Chicago, IL, USA), and HRP fluorescent substrate was purchased from Thermo Fisher Scientific. Anti-ERK1/2, -phospho-ERK1/2 (Thr202/Tyr204), -NF-κB (p65), -phospho-NF-κB (p65) (Ser536), -CDK1, -phospho-CDK1 (Thr14, 15), -AKT, -phospho-AKT (Ser473), -Bcl-2, -Bcl-xL, -Bax, -XIAP, -c-IAP1, -c-IAP2, -GAPDH, and -β-actin antibodies were obtained from Cell Signaling Technology (Danvers, MA, USA).

### 4.2. Synthesis of Test Compounds

(E)-3-(4-methoxy)-7-methoxy-4H-chromen-4-one (compound **1**, **C1**), (E)-3-(4-fluoro)styryl)-7-methoxy-4H-chromen-4-one (compound **2**, **C2**), (E)-3-(4-chloro)styryl)-7-methoxy-4H-chromen-4-one (compound **3**, **C3**), (E)-3-(4-(dimethylamino)styryl)-7-methoxy-4H-chromen-4-one (compound **4**, **C4**), (E)-7-methoxy-3-styryl-4H-chromen-4-one (compound **5**, **C5**), (E)-3-(4-(hydroxyl)styryl)-7-methoxy-4H-chromen-4-one (compound **6**, **C6,** 7M3HSC), and (E)-3-(3,4-(hydroxyl)styryl)-7-hydroxy-4H-chromen-4-one (compound A, **CA**) (Table 1) were synthesized by Knoevenagel condensation of the appropriate 3-formylchromone with selected phenylacetic acid derivatives, as described previously [61,62,63]. All the compounds were characterized by 1H NMR, MS spectra, and elemental analyses after purification by silica gel column chromatography and recrystallization. All the compounds were dissolved in DMSO at 100 mM and stored at −20 °C before use.

### 4.3. In Silico 3D Pharmacophore Analysis

In silico 3D pharmacophore search was performed with LigandScout software [78]. All compounds were constructed by Chem-Draw Professional 18.0 and converted to 3D conformations by the optimization program in LigandScout software [59,79,80]. The degrees of 3D similarity of small molecular compounds are proportional to the calculated 3D PFS values, and the 0.5 threshold (half 3D similarity) is a good choice.

### 4.4. Cells and Cell Culture

Mouse macrophage-like RAW264.7 cells and human colorectal cancer HCT116 cells were purchased from American Type Culture Collection (Manassas, VA, USA). The cells were cultured in DMEM supplemented with 10% fetal bovine serum (FBS) (Biosera Europe, Nuaillé, France) and 1% penicillin-streptomycin (Nacalai Tesque, Inc., Kyoto, Japan) in a humidified atmosphere containing 5% CO_2_ at 37 °C without antibiotics [54,55,59,60,64].

### 4.5. Assay for Cell Viability

The WST-8-based colorimetric assay was used to assess cell viability using a SYNERGY HTX multimode plate reader (BioTek Instruments, Inc., Winooski, VT, USA) according to the manufacturer’s protocols [54,55,59,60,64].

### 4.6. Assay for Cell Proliferation

Ten thousand HCT116 cells were spread to 6-well plate and cultured with **C6** and/or several DNA-damaging agents (MMS, VP-16, and CPT-11). After 48 h incubation, cells were counted under the microscopic observation using a Neubauer improved cell counting chamber (WATSON Corporation., Tokyo, Japan). The relative proliferation rate is calculated as: (count of tested group − 10,000)/(count of control group − 10,000) × 100.

### 4.7. ELISA for IL-6

RAW264.7 cells (6 × 10^4^ cells/dish) were seeded in a 96-well plate and incubated for 22 h. After changing the medium to Opti-MEM^®^, the RAW264.7 cells were pre-treated with various concentrations of compounds for 2 h and stimulated with HMGB1. After 18 h of incubation, the concentrations of IL-6 in the culture supernatants were quantified using Mouse IL-6 Uncoated ELISA Kit, in accordance with the manufacturer’s instructions [54,55,59,60,64].

### 4.8. Cell Cycle Analyses

The cell cycle was examined using the Muse^®^ Cell Cycle Kit (Luminex; Austin, TX, USA) according to the manufacturer’s instructions. Samples were analyzed using Muse^TM^ Cell Analyzer and its associated software (Luminex).

### 4.9. Caspase Activation Analyses

Caspase-3/7 activity was examined using the Muse^®^ Caspase-3/7 Kit (Luminex; Austin, TX, USA) according to the manufacturer’s instructions. Samples were analyzed using Muse^TM^ Cell Analyzer and its associated software (Luminex).

### 4.10. Trypan Blue Dye Exclusion Assay

The collected cells were resuspended in ice-cold phosphate-buffered saline (PBS). The same amount of the cell suspension and trypan blue dye (Wako Pure Chemical Industries, Ltd.; Osaka, Japan) were mixed and immediately counted under the microscopic observation by Neubauer improved cell counting chamber (WATSON Corporation., Tokyo, Japan) [54,55,56,57].

### 4.11. Western Blotting

RAW264.7 and HCT116 cells were lysed with RIPA buffer (NACALAI TESQUE, INC., Kyoto, Japan) containing a protease and phosphatase inhibitor cocktail (Roche, Basel, Switzerland). The lysates were centrifuged at 10,000× *g* for 10 min at 4 °C. The supernatants were used as cell extracts for immunoblotting analysis. Total proteins were measured by the TaKaRa BCA Protein Assay Kit (Takara Bio Inc., Kusatsu, Japan) according to the manufacturer’s instructions. The supernatant fractions were mixed with 6 × loading dye (Nacalai Tesque, Inc.), and then samples were applied to a 12.5% Extra PAGE One Precast Gel (Nacalai Tesque, Inc.). Electrophoresis was performed at a constant current of 20 mA for 90 min in running buffer (Nacalai Tesque, Inc.). Proteins were transferred to a polyvinylidene fluoride (PVDF) membrane (GE Healthcare, Chicago, IL, USA). The primary and secondary antibody reactions were performed with an iBind™ Flex Western System (Thermo Fisher Scientific Inc., Waltham, MA, USA) [54,55,59,64]. The total and phospho-forms of ERK1/2, NF-κB (p65), CDK, and AKT and IAPs (c-IAP1, c-IAP2, and XIAP), Bcl-xL, Bcl-2, and Bax proteins were detected using their rabbit antibodies (Cell Signaling Technology Inc., Danvers, MA, USA) followed by anti-rabbit HRP-labeled IgG secondary antibody (GE Healthcare). The β-actin and GAPDH proteins were detected using rabbit antibodies (Proteintech Group, Inc., Rosemont, IL, USA), followed by anti-rabbit HRP-labeled IgG secondary antibody (GE Healthcare). Chemiluminescent reactions were performed using Immobilon^®^ ECL Ultra (Merck KGaA, Darmstadt, Germany), and signals were detected by an iBright CL1000 system (Thermo Fisher Scientific Inc.).

### 4.12. EOBA

Excess-over-Bliss additivism (EOBA) provides an evaluation of the resultant cytotoxicities when two drugs are combined [66]. The formula for excess-over-Bliss is as follows:

EOBA = C − (A + B − (A × B))

where C is equal to the fractional inhibition of both drugs simultaneously, A is equal to the fractional inhibition of drug A, and B is equal to the fractional inhibition of drug B. Fractional inhibition is equal to 1.0 minus the viability (expressed as a value from 0.0 to 1.0). The near-zero EOBA scores indicate an additive (independent) drug combination. When EOBA > 0.1, it reflects a synergistic drug combination, and EOBA < −0.1 means an antagonistic drug combination.

### 4.13. Statistical Analysis

The numbers of biological and statistical significance are presented in the figure legends. Data are expressed as mean ± SE or mean ± SD. Statistical analyses were performed using the Microsoft^®^ Excel^®^ software. Student’s *t*-test was used for comparison between two variables. *p* < 0.05 was considered statistically significant. Smirnov–Grubbs test was used to remove outlier data. The distributions of cell cycle and cell status (caspase-3/7 activation) were compared by χ^2^ goodness-of-fit test. The significance level was set as α = 0.05 [54,55,59,60,64].

## 5. Conclusions

The HMGB1-RAGE signaling pathway is an important target for cancer therapeutics in relation to the TME. Here, we discovered a 3-styrylchromone derivative **C6**, which possessed both potent anti-inflammatory and anti-cancer activities by blocking the HMGB1-RAGE-ERK1/2 axis. ERK1/2 regulates not only inflammation but also cell proliferation and apoptosis. This study demonstrated that **C6** potently inhibited RAGE-mediated ERK1/2-NF-κB activation to induce pro-inflammation in macrophage-like RAW264.7 cells. On the other hand, in HMGB1-stimulated HCT116 cancer cells, **C6** was revealed to suppress the activation of ERK1/2, CDK1, and AKT and the down-regulation of anti-apoptotic XIAP but not c-IAP1, c-IAP2, and Bcl-xL and up-regulate pro-apoptotic Bax and caspase-3/7. Furthermore, drug combination strategies of **C6** and DNA-damaging agents (MMS, VP-16, and CTP-11) were shown to be synergistically effective for cancer cell treatment. Taken together, the results of this study indicate that the reduction of anti-apoptotic XIAP and the induction of pro-apoptotic Bax are important mechanisms of the novel combination of **C6** and DNA-damaging agents to induce apoptosis to cancer cells. This combination may represent an option for a new type of cancer therapy. Furthermore, the understanding of the linking of carcinogenesis and inflammation in the TME by using **C6** could translate into therapeutics that target tumor-supporting immune cells as well as cancer cells.

## Figures and Tables

**Table 1:** General structural units of 3-styrylchromone derivatives and their anti-inflammatory activities.

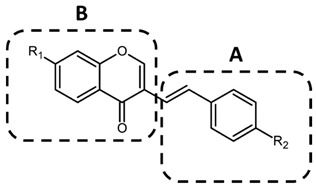			
Compound	R1	R2	IL-6 Production Inhibition EC50 (μM) *
**C1**	–OCH_3_	–OCH_3_	11.1 ± 7.63
**C2**	–OCH_3_	–F	>10
**C3**	–OCH_3_	–Cl	>10
**C4**	–OCH_3_	–N(CH_3_)_2_	>10
**C5**	–OCH_3_	–H	>10
**C6**	–OCH_3_	–OH	0.018 ± 0.025
**C7**	–H	–OH	0.020 ± 0.016

* The suppression of IL-6 production by treatment with 3-styrylchromone derivatives in HMGB1-stimulated RAW264.7 cells was measured by ELISA as described in Section 4. The EC50 values were calculated using the titration curves (Figure 1b and Figure 2b) and are presented as mean ± SE of three independent experiments.

## Data Availability

Not applicable.

## Supplementary Materials

The following supporting information can be downloaded at the following: https://www.mdpi.com/article/10.3390/ijms23073426/s1.
