# Supplementary material for: A Dual Anti-Inflammatory and Anti-Proliferative 3-Styrylchromone Derivative Synergistically Enhances the Anti-Cancer Effects of DNA-Damaging Agents on Colon Cancer Cells by Targeting HMGB1-RAGE-ERK1/2 Signaling
Source: Int J Mol Sci. 2022 Mar 22;23(7):3426. doi: 10.3390/ijms23073426 (PMC8998738; doi:10.3390/ijms23073426)
Supplement: Supplementary file 1 [file ijms-23-03426-s001.zip › ijms-1633728-supplementary.pdf]

International Journal of *Molecular Sciences*

MDPI

Supplementary Materials

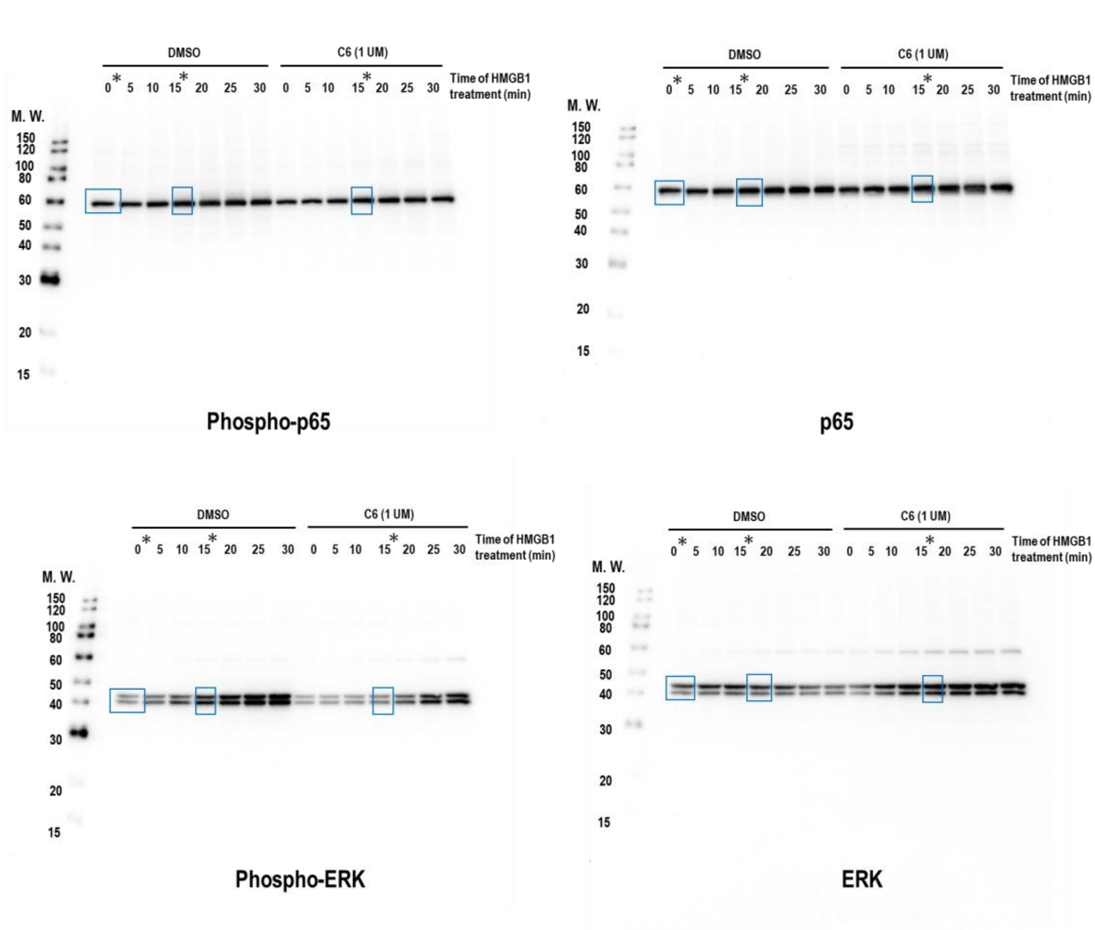

n=1

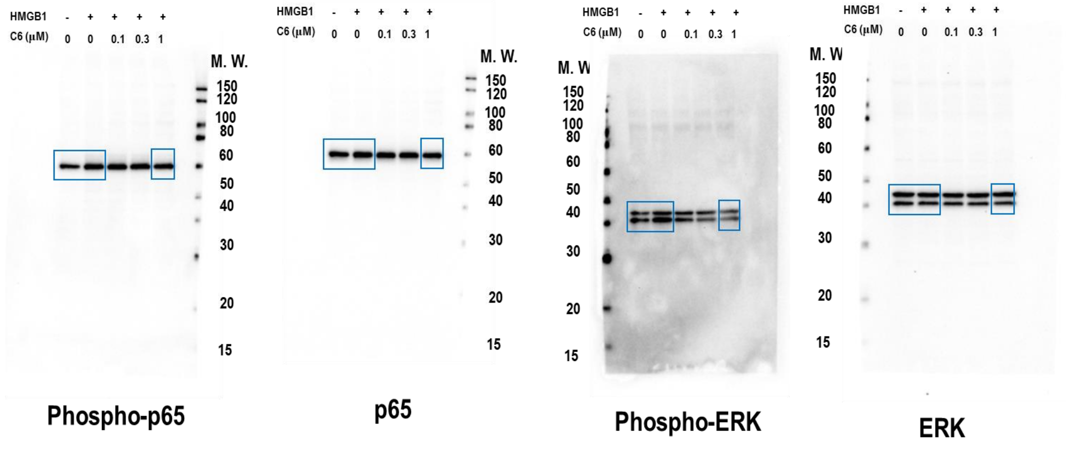

n=2

n=3

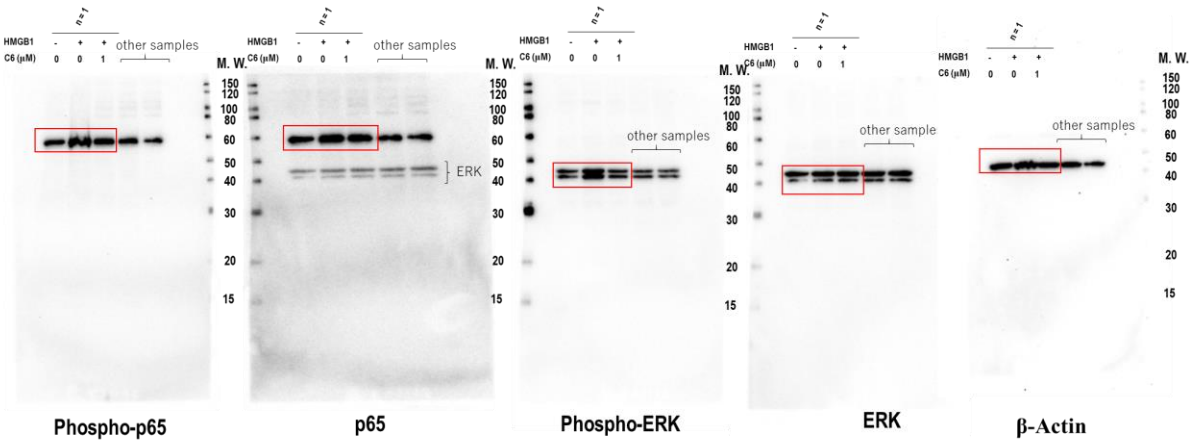

Figure S1. The whole membranes and cropped regions of the immunoblot panels in Figure 2c. Data for immunoblot analyses were obtained 3 times. RAW 264.7 cells were pre-treated with **C6** for 2 h and then stimulated with HMGB1 (5 μg/mL) for the indicated times. Cell lysates were subjected to Western blot analyses using primary anti- phospho-ERK1/2, -total-ERK1/2, -phospho-p65, and –total-p65 antibodies. β-Actin was checked for one part of membrane (n=3). The blue rectangles show the bands of interest, and red rectangles show the representative bands used in the figure. In the first membrane, cells were collected every 15 min up to 30 min after HMGB1 treatment with or without 1 μM **C6**. Second, 0.1, 0.3, and 1 μM C6 was treated with or without HMGB1 and collected at 15 min. Third, 1 μM **C6** was treated with or without HMGB1 and collected at 15 min.

n=1

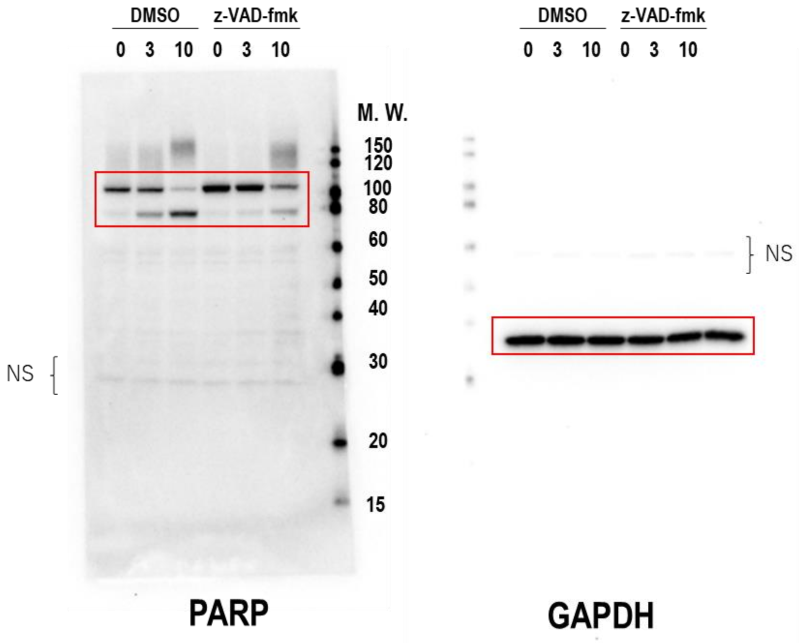

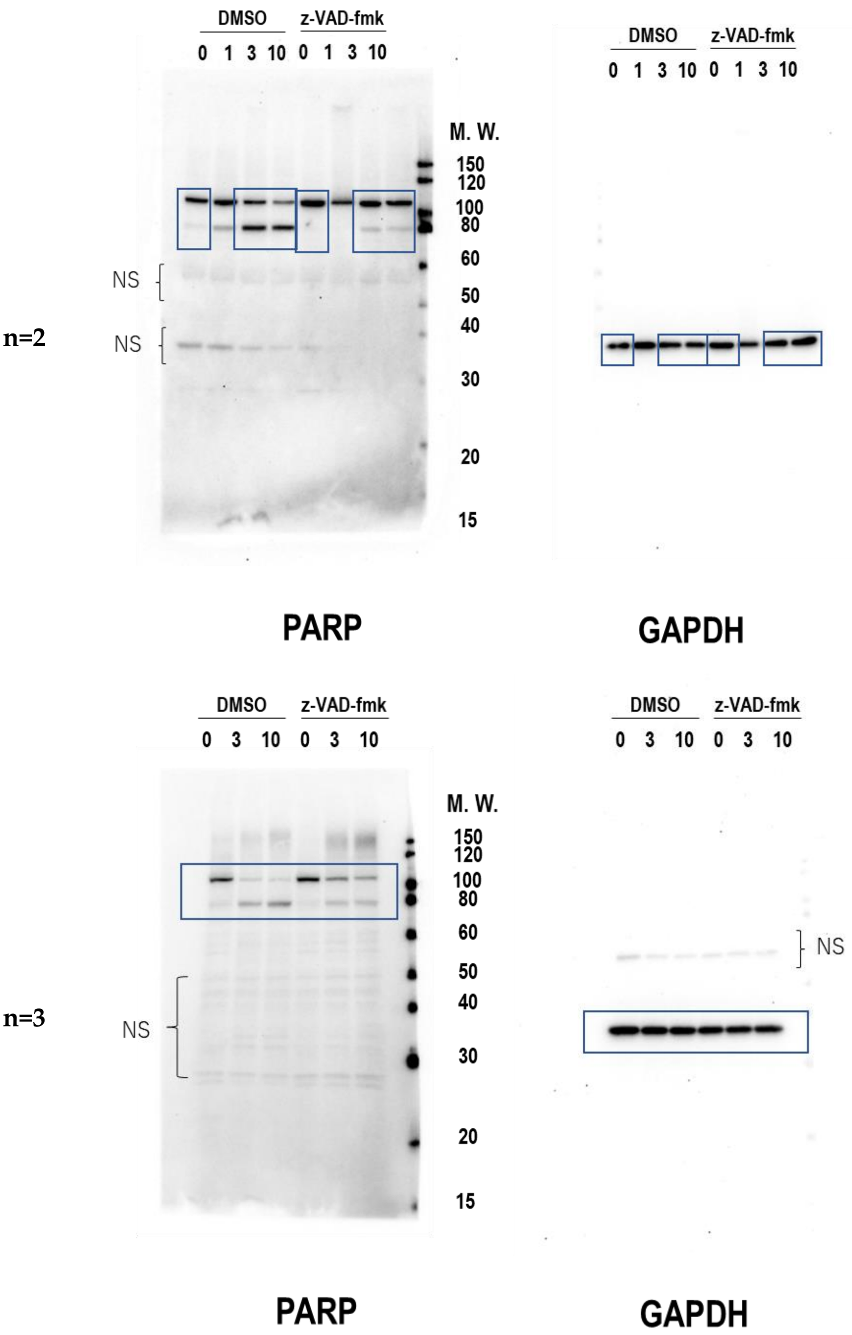

Figure S2. The whole membranes and cropped regions of the immunoblot panels in Figure 4c. Data for immunoblot analyses were obtained 3 times. HCT116 cells were pre-treated with or without 20 µM z-VAD-fmk for 2 h, and followed by treatment with 0, 3, and 10 µM of **C6** for 48 h. The blue rectangles show the bands of interest, and red rectangles show the representative bands used in the figure. NS, non-specific bands.

Phospho-ERK

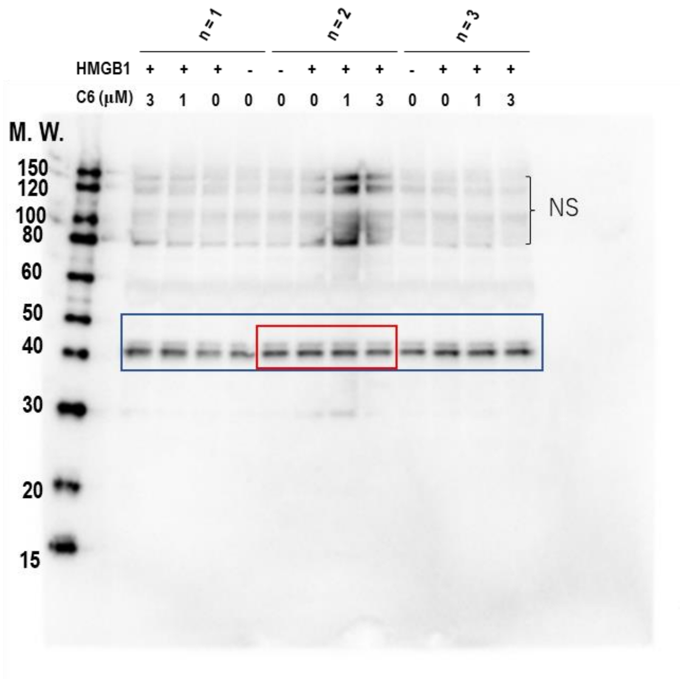

Total ERK

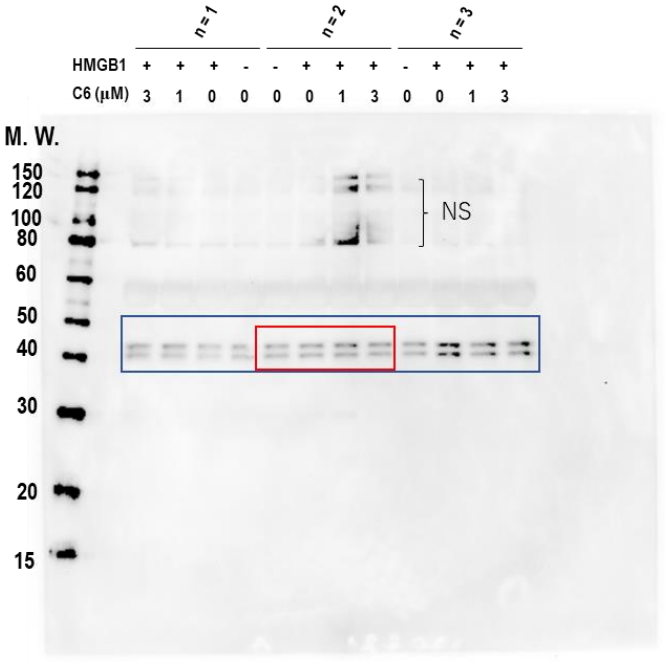

GAPDH

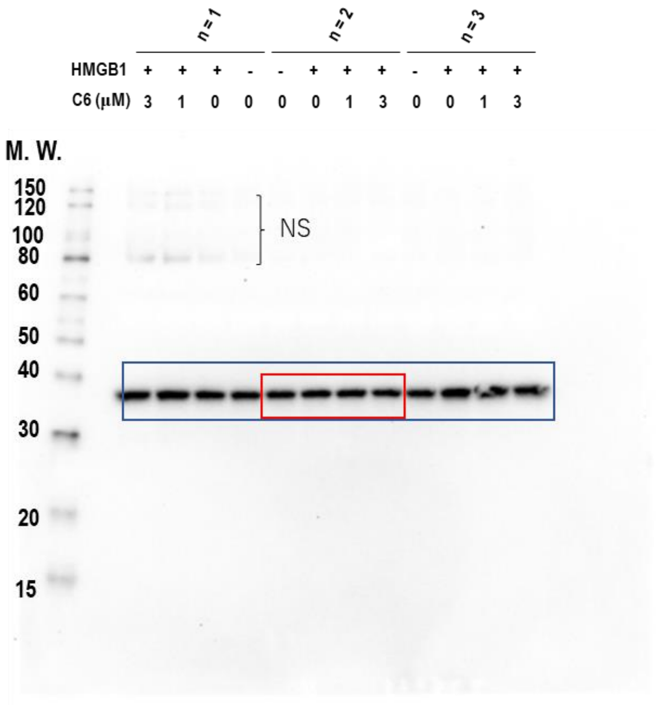

Figure S3. The whole membranes and cropped regions of the immunoblot panels in Figure 5b. Data for immunoblot analyses were obtained 3 times. HCT116 cells were pre-treated with or without 1 and 3 μM **C6** followed by 10 μg/mL HMGB1 for 30 min. The blue rectangles show the bands of interest, and red rectangles show the representative bands used in the figure. NS, non-specific bands.

Phospho-Akt & total-Akt

1. DMSO
2. 10 μg/mL HMGB1
3. 10 μg/mL HMGB1 + 1 μM C6

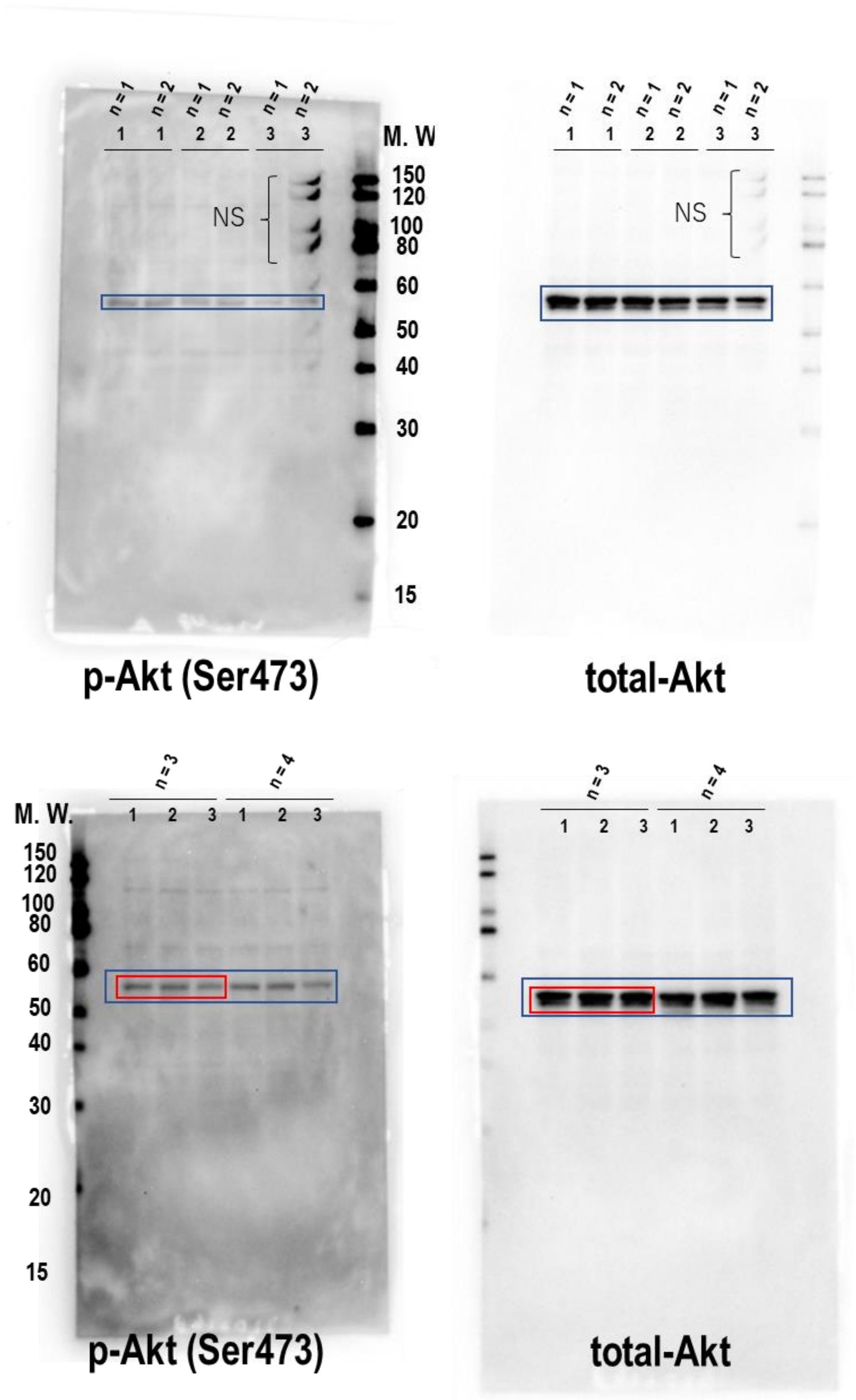

Figure S4. The whole membranes and cropped regions of the immunoblot panels in Figure 6. Data for immunoblot analyses were obtained 4 times. HCT116 cells were pre-treated with or without 1 μM **C6** followed by 10 μg/mL HMGB1 for 72 h. The blue rectangles show the bands of interest, and red rectangles show the representative bands used in the figure. NS, non-specific bands. Stripping was done by WB Stripping Solution (nakalai tesque, Kyoto, Japan), but sometimes remains former bands such as GAPDH in XIAP and CDK1 in c-IAP2 images.

Phospho-CDK1 & total-CDK1

1. **DMSO**
2. **10 μg/mL HMGB1**
3. **10 μg/mL HMGB1 + 1 μM C6**

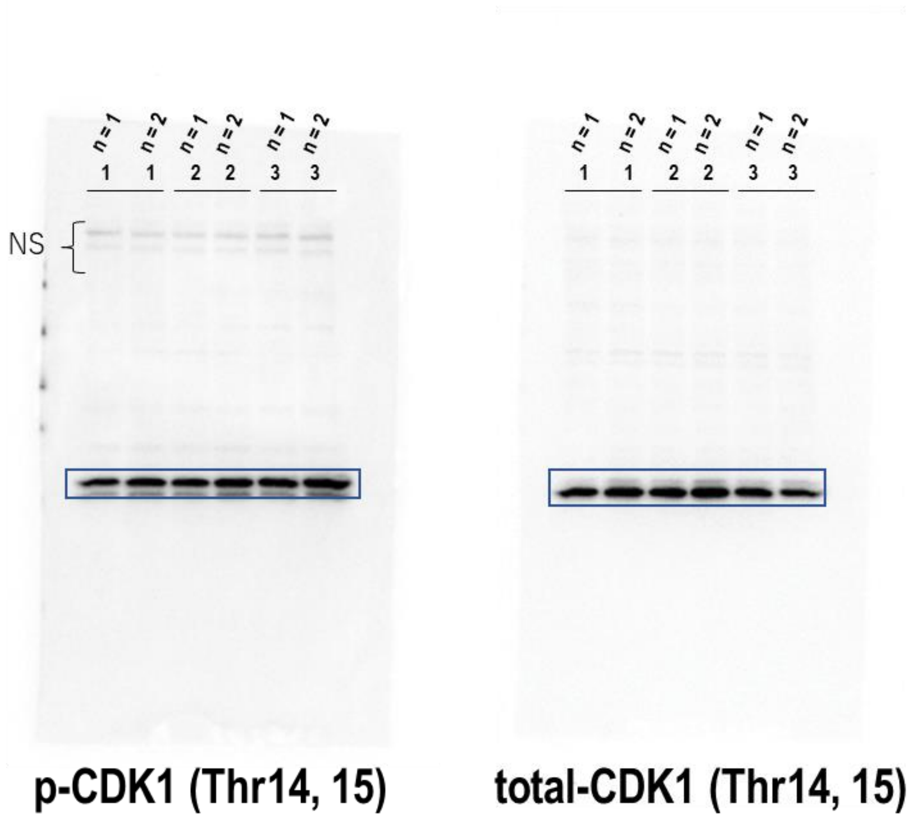

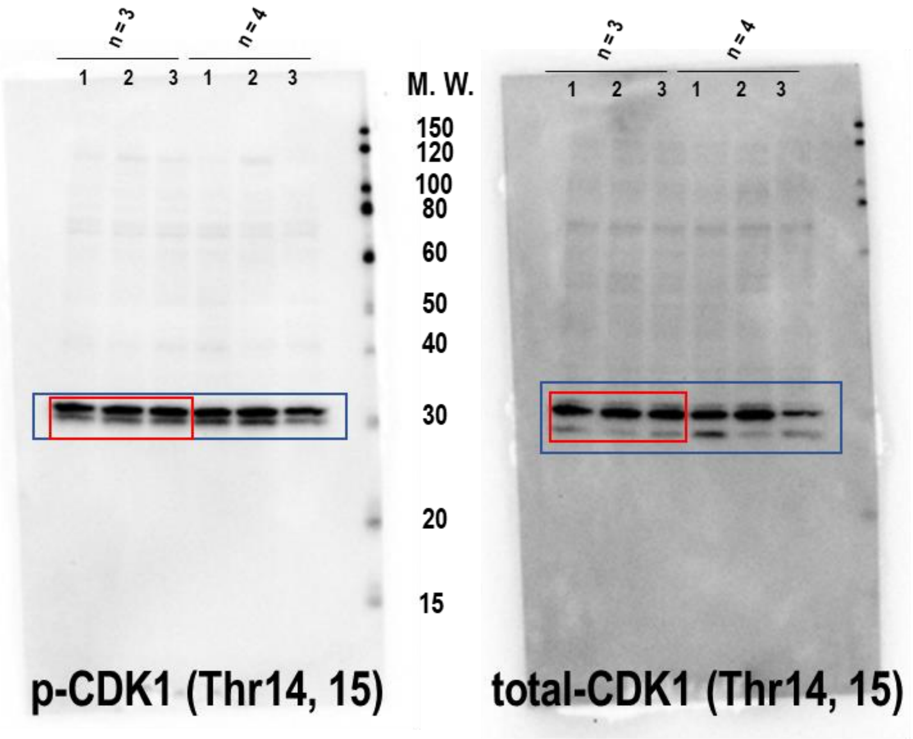

1. DMSO
2. 10 μg/mL HMGB1
3. 10 μg/mL HMGB1 + 1 μM C6

c-IAP1

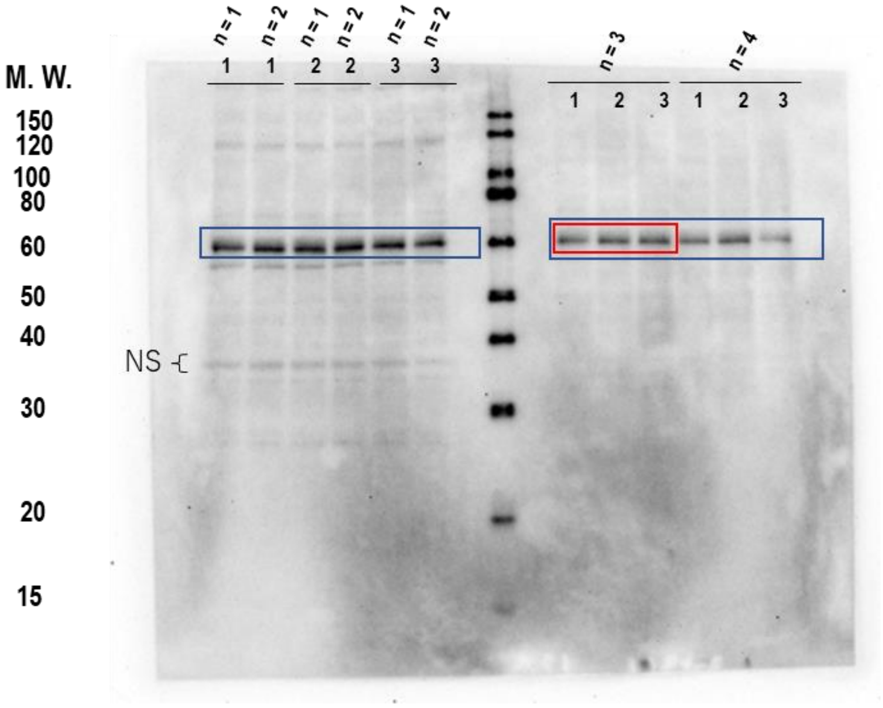

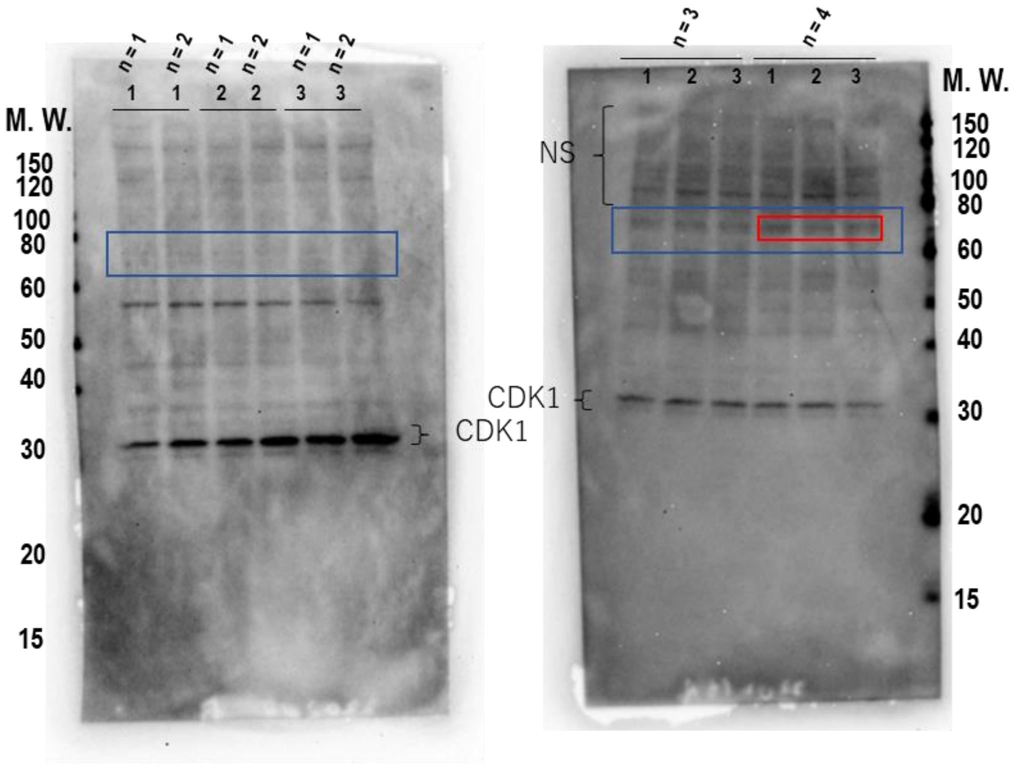

c-IAP1

c-IAP2

1. **DMSO**
2. **10 μg/mL HMGB1**
3. **10 μg/mL HMGB1 + 1 μM C6**

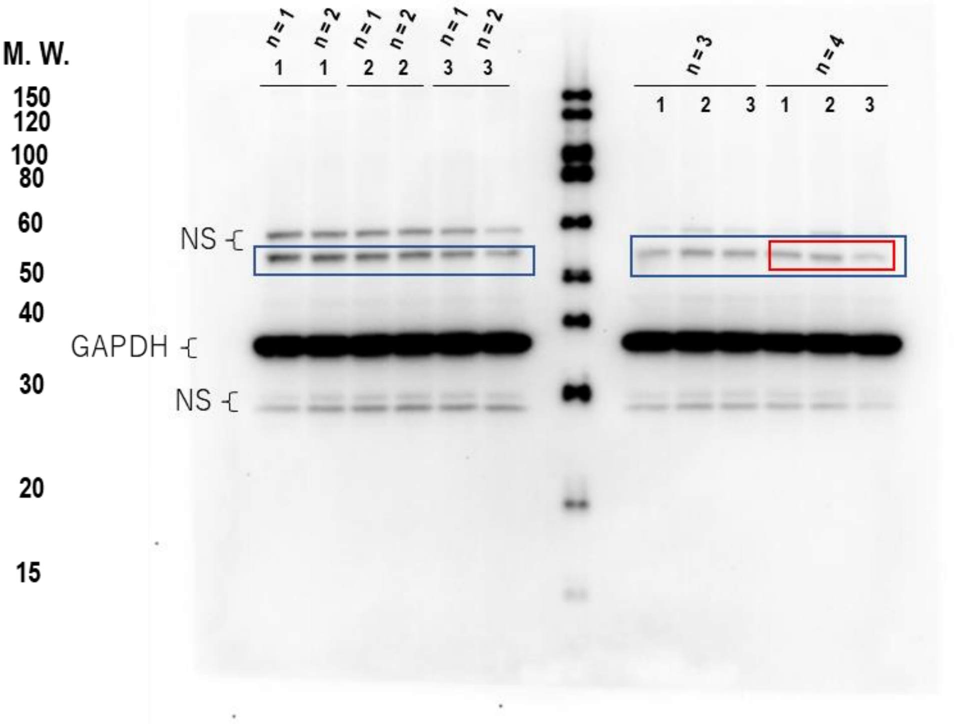

XIAP

GAPDH

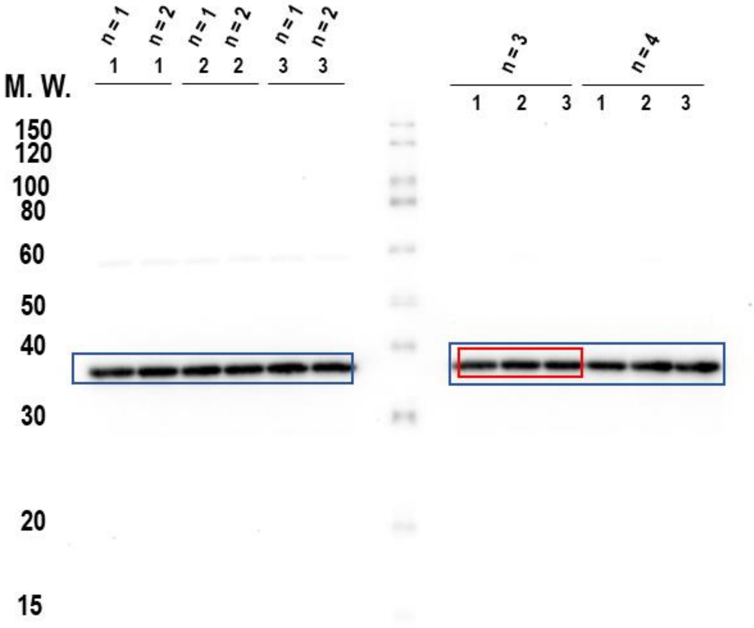

1. **DMSO**
2. **10 μg/mL HMGB1**
3. **10 μg/mL HMGB1 + 1 μM C6**

Bcl-xL

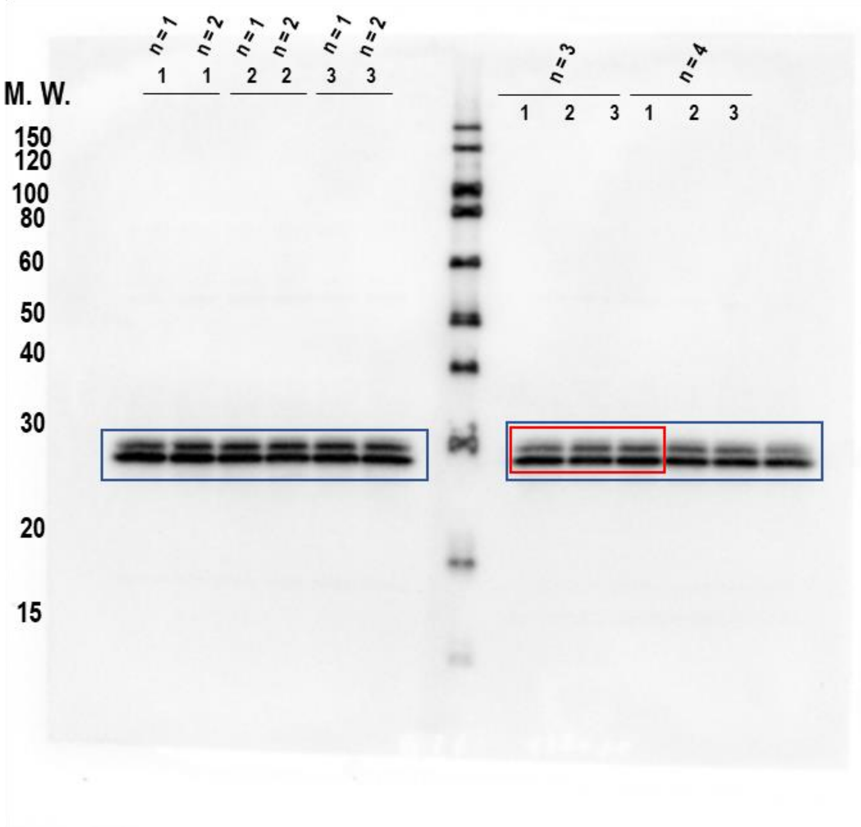

Bax

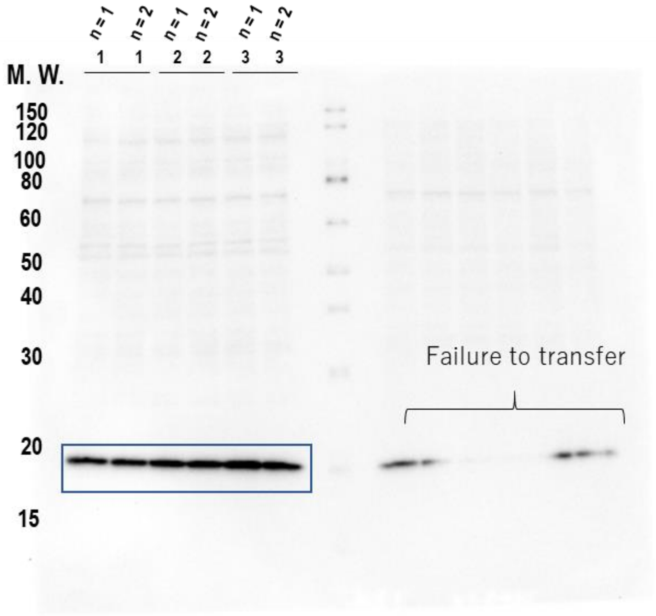
n = 1
n = 2
n = 1
n = 2
n = 1
n = 2
1
1
2
2
3
3
M. W.
150
120
100
80
60
50
40
30
20
15
Failure to transfer

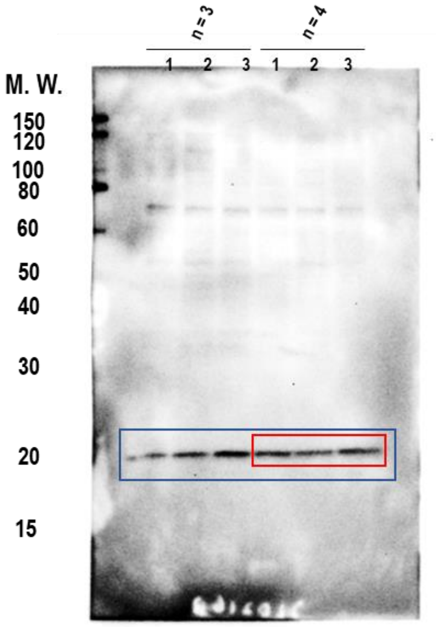
n = 3
n = 4
1
2
3
1
2
3
M. W.
150
120
100
80
60
50
40
30
20
15
